# Accelerating upward treeline shift in the Altai Mountains under last-century climate change

**DOI:** 10.1038/s41598-019-44188-1

**Published:** 2019-05-22

**Authors:** Roberto Cazzolla Gatti, Terry Callaghan, Alena Velichevskaya, Anastasia Dudko, Luca Fabbio, Giovanna Battipaglia, Jingjing Liang

**Affiliations:** 1 Department of Forestry and Natural Resources, Purdue University, West Lafayette, USA; 2 Biological Institute, Tomsk State University, Tomsk, Russia; 3 Department of Animal and Plant Sciences, University of Sheffield, Sheffield, UK; 4 Department of Biological, Geological and Environmental Sciences (DBGES), University of Catania, Catania, Italy; 5 Department of Environmental, Biological, and Pharmaceutical Sciences and Technologies (DiSTABiF), University of Campania “Luigi Vanvitelli”, Caserta, Italy

**Keywords:** Phenology, Climate change

## Abstract

Treeline shift and tree growth often respond to climatic changes and it is critical to identify and quantify their dynamics. Some regions are particularly sensitive to climate change and the Altai Mountains, located in Central and East Asia, are showing unequivocal signs. The mean annual temperature in the area has increased by 1.3–1.7 °C in the last century. As this mountain range has ancient and protected forests on alpine slopes, we focus on determining the treeline structure and dynamics. We integrated *in situ* fine-scale allometric data with analyses from dendrochronological samples, high-resolution 3D drone photos and new satellite images to study the dynamics and underlying causal mechanisms of any treeline movement and growth changes in a remote preserved forest at the Aktru Research Station in the Altai Mountain. We show that temperature increase has a negative effect on mountain tree growth. In contrast, only younger trees grow at higher altitudes and we document a relatively fast upward shift of the treeline. During the last 52 years, treeline moved about 150 m upward and the rate of movement accelerated until recently. Before the 1950s, it never shifted over 2150–2200 m a.s.l. We suggest that a continuous upward expansion of the treeline would be at the expense of meadow and shrub species and radically change this high-mountain ecosystem with its endemic flora. This documented treeline shift represents clear evidence of the increased velocity of climate change during the last century.

## Introduction

Over the last century, planet Earth has experienced significant temperature increases, particularly in the Northern Hemisphere^1,2^. Meanwhile, more climate variability is predicted with a general increase in temperature and extreme dry and hot periods^3,4^. Such events have a substantial effect on montane forest ecosystems^5,6^, where rising temperatures enable many tree species to establish at higher elevations for new and favorable environmental niches^7–9^, and other better growing conditions^10–12^.

There is clear evidence that temperate and boreal forests are encroaching northwards and upwards into tundra and alpine communities^13–15^, and such climate-driven effects may cause a treeline shift that changes the structure of montane and tundra systems^16^. For instance, a long-term study in Scandinavia^17^ has shown an upward movement of treeline in the order of 150–200 mover about 30 years. In temperate regions, tree species have been shown to migrate naturally by several kilometers a century^18,19^. However, rapid climatic changes can lead to a 100–1000 times faster shift^20^, as demonstrated by a long-term photographic record has been used to document the higher altitude shift of alpine treeline into alpine meadows in Yunnan, China^21^. In North America, modelling of tree species distributions in relation to climate change suggests a general movement of 134 eastern tree species ranges towards the Northeast^22^. In most cases, these changes have been linked to an observed general warming. However, treeline location and dynamics have complex causes^23^ and over the past 100 years, treeline in northern Sweden increased in altitude, decreased and remained stable on neighboring mountains all experiencing the same climatic warming^24^. Controls on treeline include temperature, herbivory, lack of niches for seedling establishment, etc.^25^.

The Altai Mountains, located in Central and East Asia, are showing evident impacts of the changing climate, including significant changes of ecological succession in the areas emerged from retreating glaciers^26–28^. Although no relevant variations in the mean annual precipitation have been reported for the area^29^, the mean annual temperature in the Northern Altai Mountains has shown a significant increase of 1.3–1.7 °C in the last century^30^. This general trend was also confirmed by a comprehensive study that analyzed 50 years of temperature and precipitation variations in the whole mountain chain in both Russia and China^31^.

Several studies reconstructed the impact of climate change in the Central Eurasia from tree ring-based records^32–39^. These studies in the Tianshan and Altai Mountains and in other high-mountains of the world^40,41^, showed that climate change might have a harmful effect on tree growth and recent evidence reported that the lower forests can also display “browning” over extensive areas in the boreal region^42^. The treeline expansion at these latitudes, such as in Siberia, may lead to a compositional change of forest communities and could result in a significant alteration of biotic interactions^43^. Meanwhile, several studies showed that slightly higher temperatures and greater accumulation of carbon dioxide in the atmosphere accelerate the growth rate of species in forest ecosystems^44,45^.

Treeline shift and changes in tree growth^46^ are evident signs of various aspects of global climatic changes^47^ and some regions are more sensitive to the global climate changes than other areas of the world^21^.

Remote sensing and aerial photographs are typically used to assess the movement of treeline in high mountains over the past 30 years^48^, and ground measurements and repeat photography over the past 100 years^24^. However, for most remote areas especially in the Altai Mountains, especially in the Russian part, the absence of aerial photographs combined with a lack of satellite images before the 1980s has impeded an understanding of the effects of climate change on treeline. In particular, for the Russian Altai Mountains, only scarce and sporadic climatic observations are available before the mid-1900s and historical photographic documentation (in terms of both ground/aerial pictures and satellite images) are scarce.

Here we integrated *in situ* fine-scale allometric data, dendrochronological samples, high-resolution 3D drone photos and new satellite images to characterize the treeline, with the aim to look at the recent dynamics and growth changes and to construct a baseline to follow future changes, in a remote preserved forest at the Aktru Research Station in the Altai Mountains.

## Study Area

The Aktru Research Station is located in the Southeastern Altai Republic (Russia) close to the borders of Mongolia and China in the Central Eurasian Continent (50°06′03″N, 87°40′14″E). With an altitude of 2100 m a.s.l., the station is situated in the high alpine range of these mountains. There are no major anthropogenic disturbances on the surrounding ecosystems and vegetation, since the area is located in a very remote (with a 3-day drive from the closest airport) and a high-altitude part of the Asian continent. The nearest settlement (Kuray, a small village) is located downstream from the station at about 30 km southeast and the nearest town is Gorno-Altaisk, about 250 km northwest of the station. The Aktru Research Station was founded in 1956 by V.M. Tronov and has been in operation until today under the supervision of Tomsk State University. There is no evidence or documentation on any type of logging, big herbivore (wild or domesticated) grazing, or fire-related impact, at least for the last 50 years, on the vegetation of the mountainside where our study site is situated.

**Figure 1:**
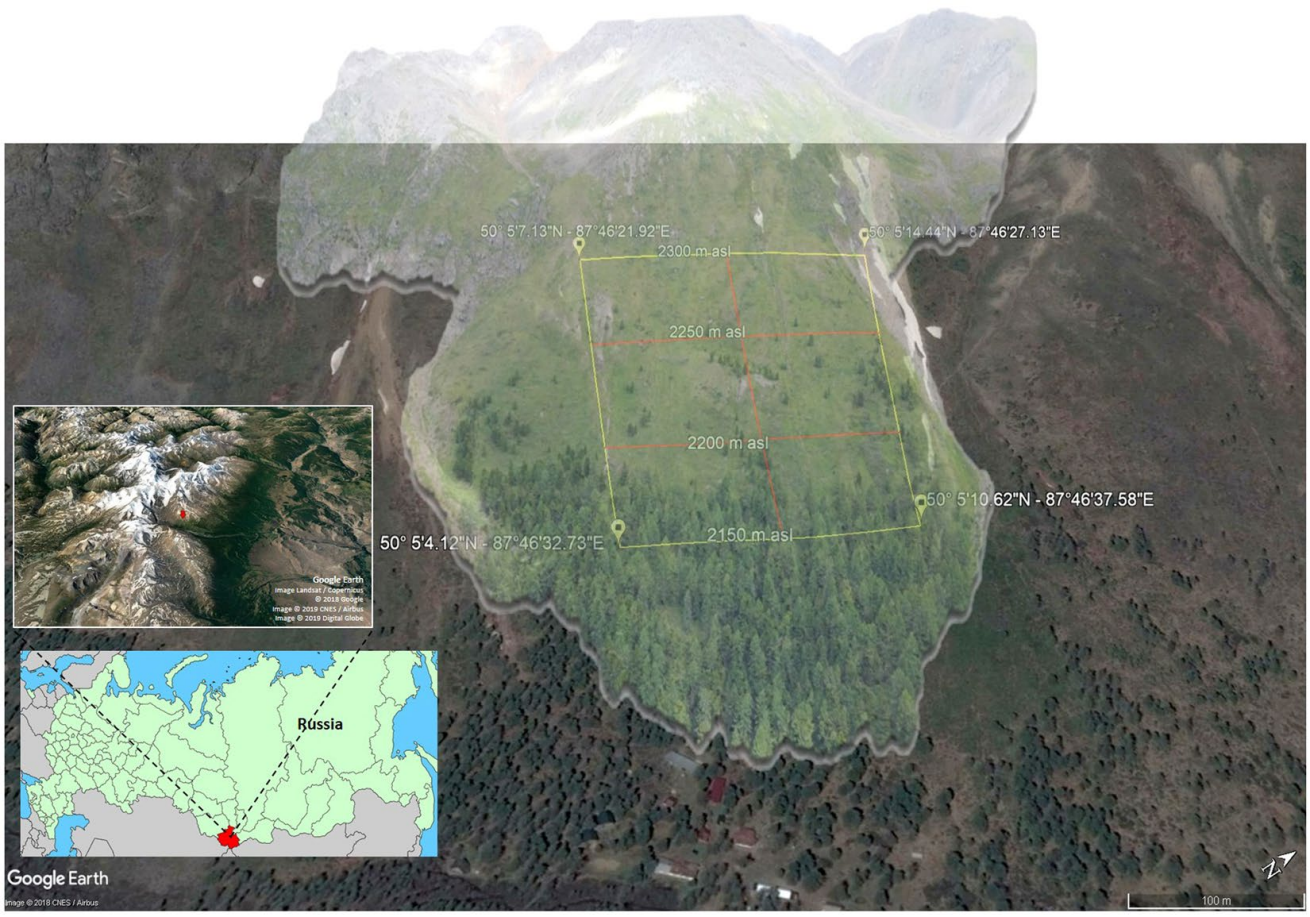
A 3-D model (built by meshing 10 aerial photographs taken by a drone) of the study site on the mountains slope above the Aktru Research Station in the Altai Mountains of Russia (small inset). The model is overlaid on the most recent available (2017) satellite image of the area to show the sampling scheme composed of three vertical and four horizontal transects, placed at a 50-m distance. The geographic coordinates at the corner of the study sites and the altitudes are shown. The smaller inset shows the continuous forest belt between the Severo-Chuyskiy Mountain Chain (white mountaintops on the left) and the Kuray Valley (sandy terrain on the right). The highest altitudinal treeline level is reached at the study site (red arrow) of the Aktru Research Station (inset orientation is the same as the larger satellite image; map data of the inset: Google Earth, Landsat/Copernicus, CNES/Airbus, Digital Globe). The original satellite imagery, obtained from Google Earth Pro© version 7.3.2.5491 (https://www.google.com/earth/; map data: Google, CNES/Airbus), was modified with MeshLab and Sketchup software.

The study site (Fig. 1) is located on the northwestern slope of the Severo-Chuyskiy Mountain Chain, just above the research station, at an altitude between 2150 and 2300 m a.s.l. The climate of the area is characterized by low temperatures (annual average −5.2 ± 1.9 °C, summer average +8.7 ± 2.3 °C), high average annual precipitations (500–800 mm), and high diurnal temperature variation (of 15–20 °C)^29^. The site is considered to be climatically representative of the Altai, and includes several mountain peaks and glaciers^29^. The slope of this mountainside is 43.4–54.1%.

## Results

**Figure 2:**
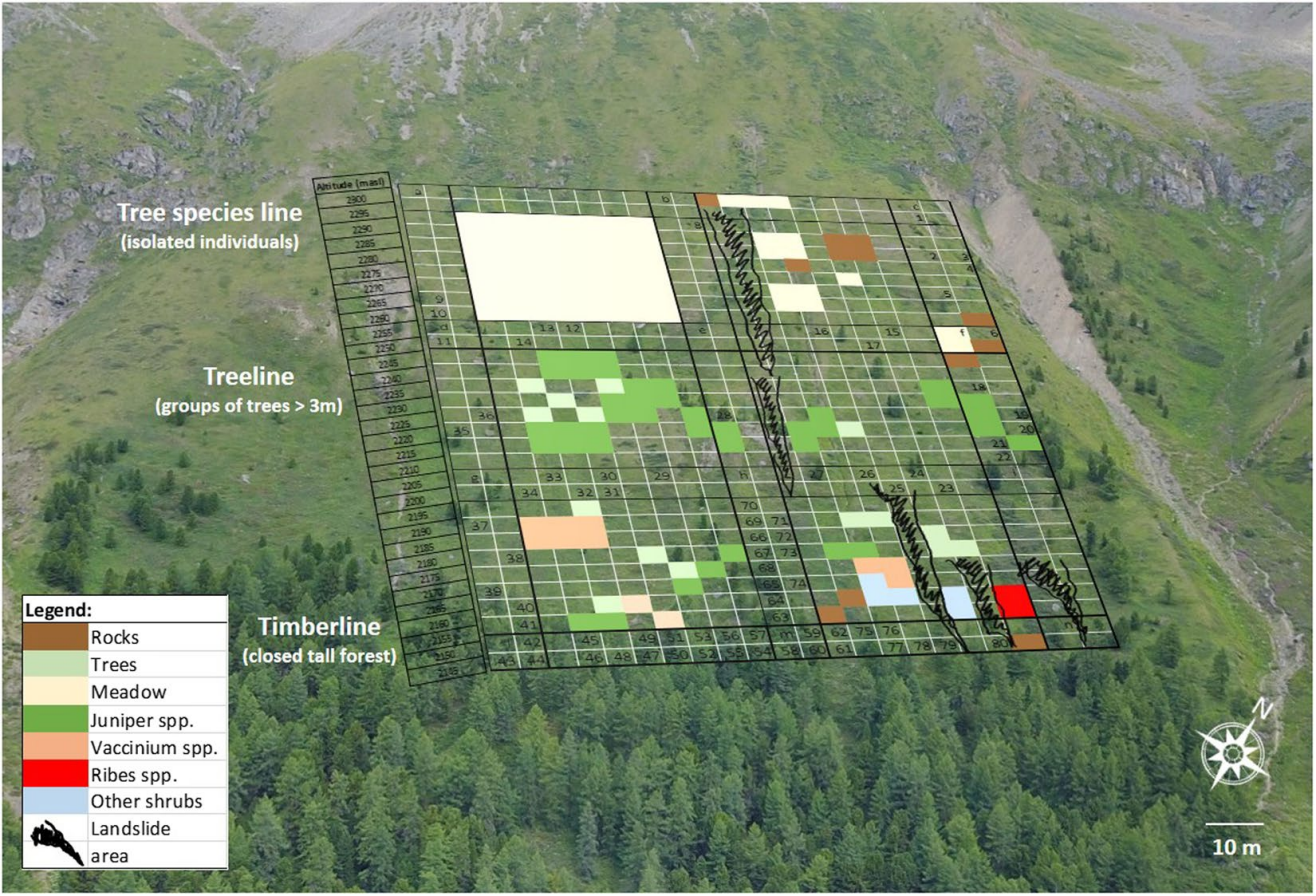
A graphical representation of the main vegetation detected in the field on a fine-scale sampling grid of 5 × 5 m. Numbers in the main transects (three vertical and four horizontal) represent tree IDs whose dimensions (height, d.b.h., crown width) and species have been recorded for all the trees with d.b.h. >1 cm. The legend explains the vegetation and geomorphological representation on the grid with an altitudinal scale placed on the left side of the sampling grid. The background aerial-photo was taken with a drone. Labels at the elevation of the timberline, treeline and tree species line (definitions are in ref.^49^) are displayed.

The densest timberline in the study site, composed by two species (*Pinus siberica* 50–60%, *Larix siberica* 40–50%), is located up to an altitude of ≈2150 m a.s.l. (Fig. 2). Above this level (approximately between 2150 and 2250 m a.s.l.), a treeline composed of sparse groups of trees (belonging to the same two species of the treeline) and shrubs (mostly of *Juniper spp*., *Vaccinium spp*. and *Ribes spp*.) were detected. From 2250 m up to 2300 m a.s.l., only isolated trees and herb-rich meadows were identified (tree species line). No trees or shrubs with a d.b.h. >1 cm (see methods) were detected above ≈2300 m a.s.l. A few landslides and groups of rocks were located on the northern side of the treeline area. The whole study site (Fig. 2) from 2150 m to 2300 m a.s.l. may be defined, according to the common scientific convention^49^, as a “high-altitude treeline ecotone”.

**Figure 3:**
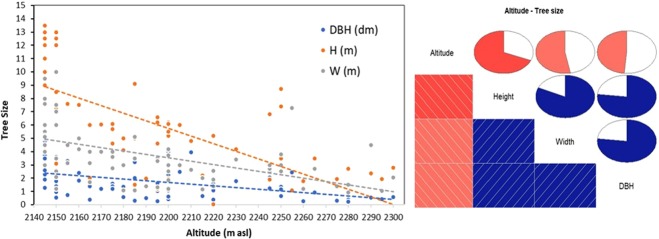
The behavior of allometric parameters (DBH = diameter at the breast height; H = height; W = crown width) along incremental altitude on the treeline ecotone. Left: scatter plot with tendencies lines (correlation coefficients are reported in the text). Right: the correlogram between the altitude and the tree size parameters, in which the lines and color of the boxes indicate the slope and the sign of the correlation, the pie charts represent the value of the Spearman’s correlation index.

As expected, we found a significant decline in tree size towards higher altitudes (Fig. 3). In particular, tree height had the highest negative correlation with the altitude (Spearman’s rank correlation ρ = −0.76, P < 0.01), followed by the crown width (ρ = −0.66, P < 0.01) and d.b.h. (ρ = −0.54, P < 0.01). No trees higher than 6 m where found over 2250 m a.s.l., even though a few relatively tall trees (7–9 m) were able to live at an altitude of 2240–2250 m a.s.l.

**Figure 4:**
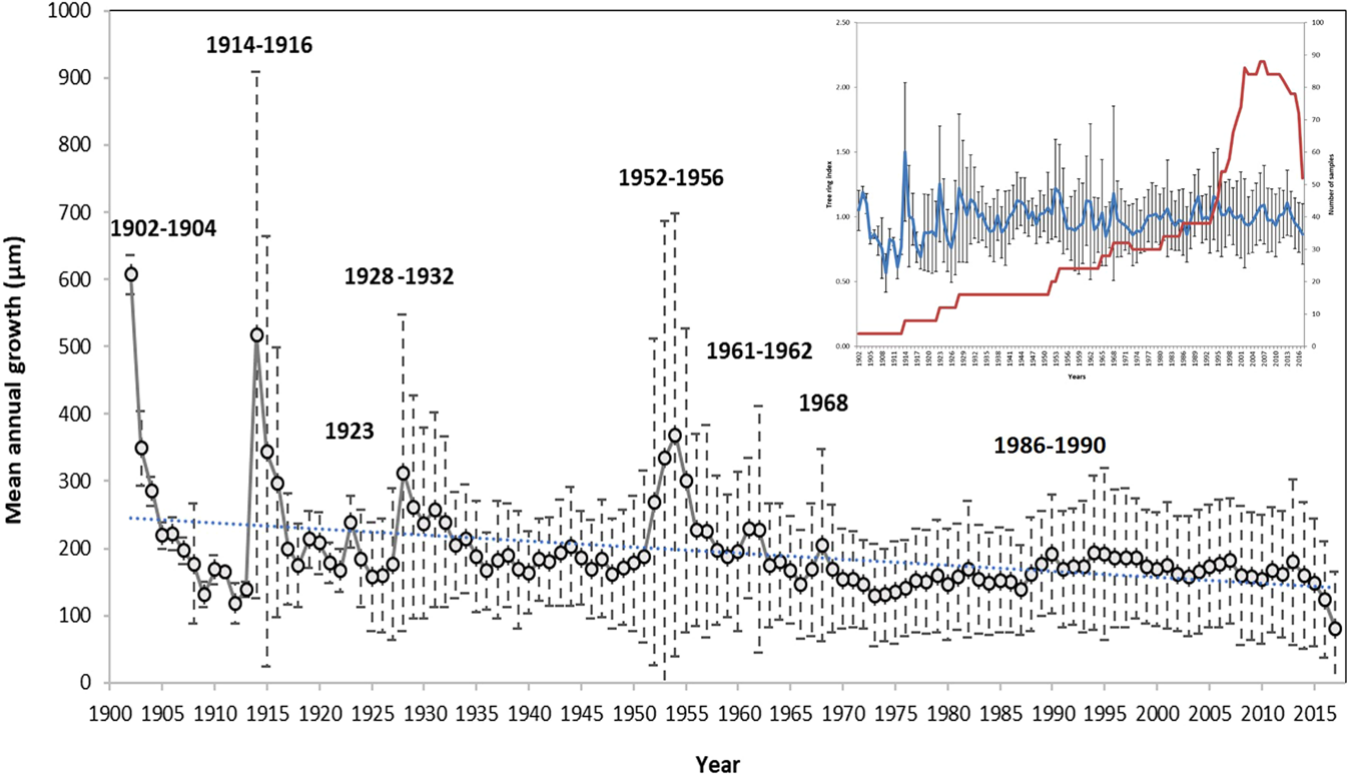
The complete dendrochronological curve calculated as the mean annual growth from the 48 wood core samples collected along the altitudinal transects. The years for the relevant peaks are indicated above the high growth points. The dashed line represents the linear tendency and buffers the standard deviation (detailed statistical analysis in Fig. 5). In the inset, the tree ring index calculated accounting for the autocorrelation and partial autocorrelation functions is shown as a time series (blue line) and plotted together with the number of collected samples (red line) against the increasing years (in abscissa).

The dendrochronological analyses of the 48 wood cores collected from different altitudes along the transect of the treeline ecotone, shows a slightly declining mean annual growth curve between 1902 and 2017, with some periods (1902–1904, 1914–1916, 1923, 1928–1932, 1952–1956, 1961–1962, 1968) that peaks above the annual average tendency (Fig. 4). However, no relevant peaks are detected after the year 1968 (i.e. during the last 50 years of the dendrochronology).

**Figure 5:**
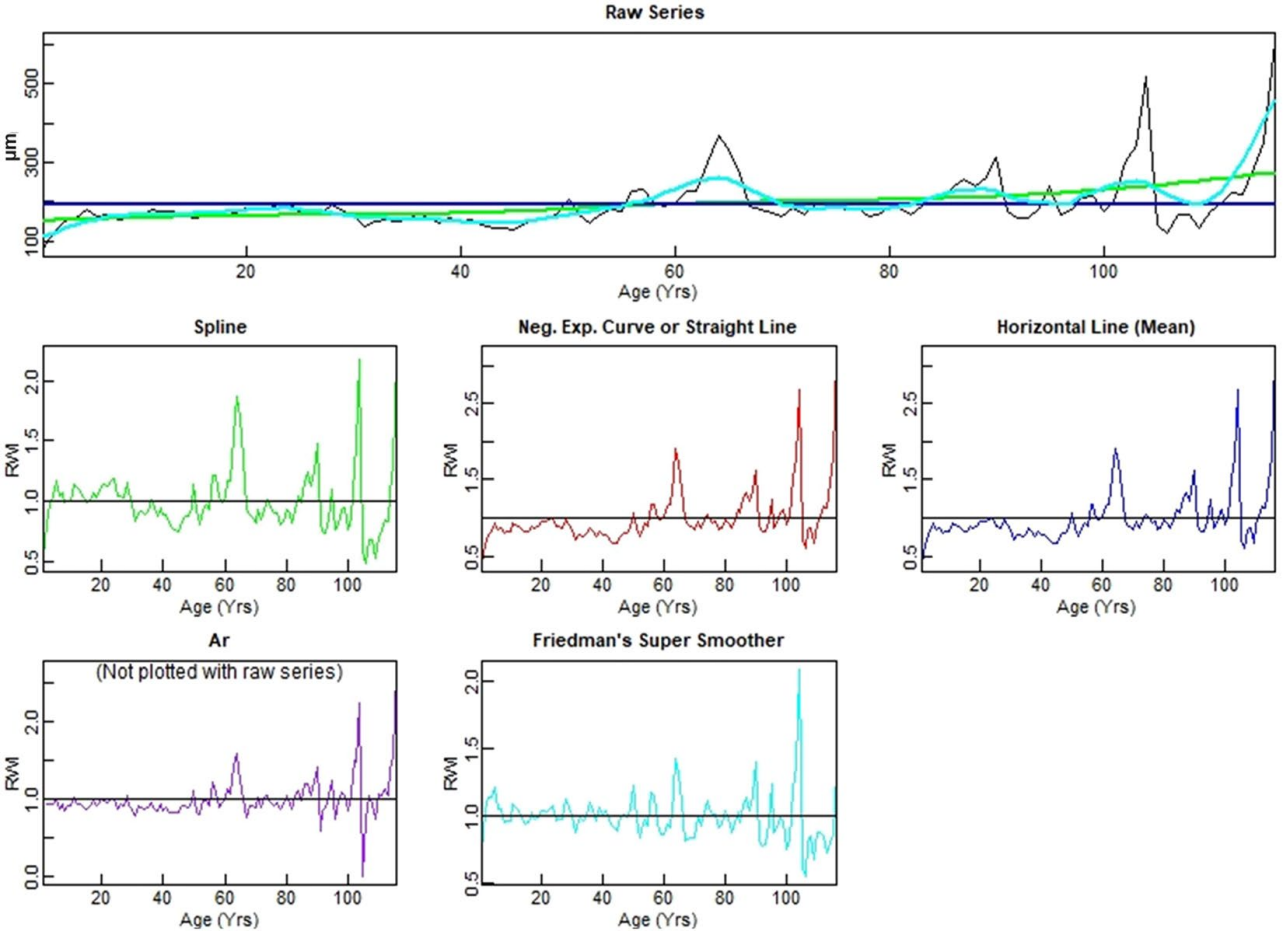
The raw time-series (highest plot) is detrended and standardized by five methods. The detrended curve shown accounts for the tree’s natural biological growth trend. Except for the Ar, the other four detrended curves are plotted over the raw series and show a decreasing growth trend, particularly after 60 years. Notice that the x-axis here is the opposite to that in Fig. 4 (showing the curve increasing from the oldest to the youngest age, i.e. the abscissa represents the age of the tree while in Fig. 4 it represents the year of birth) and should be chronologically read from right to left.

After normalizing the time-series data with different methods (Fig. 5), the detrended curves (Spline and Friedman’s Super Smoother in comparison with Straight and Horizontal Lines) show similar peaks with respect to the raw curve (in Fig. 4) and a general decreasing growth trend, as confirmed by the Mann-Kendall statistics (τ = −0.315, P < 0.01).

**Figure 6:**
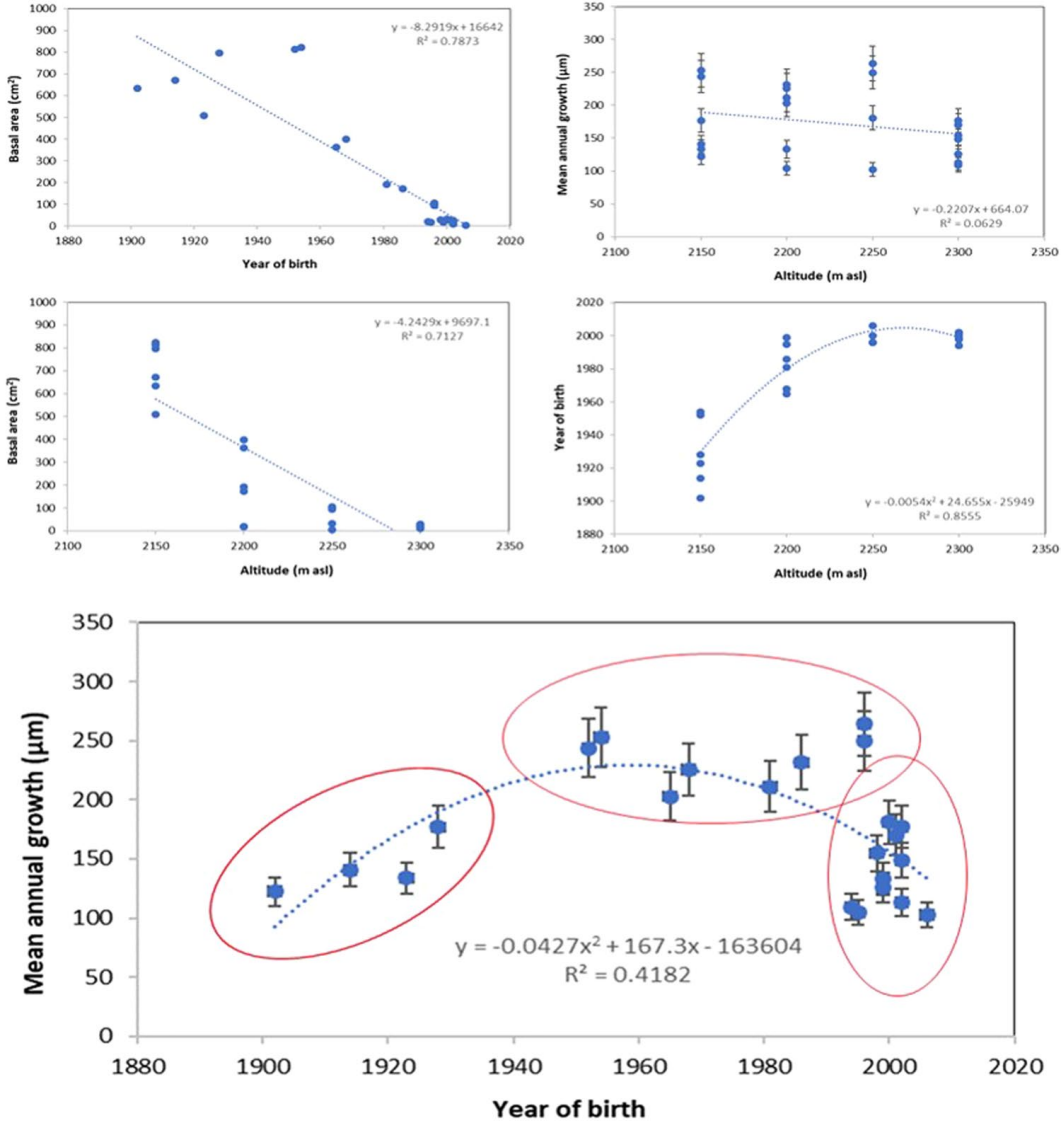
Scatter plots between basal area, mean annual growth, year of birth and altitude. Linear or quadratic equations and coefficients of determination are shown in each plot. The plot at the bottom shows young, mid-age and old trees grouped according to their mean annual growth.

**Figure 7:**
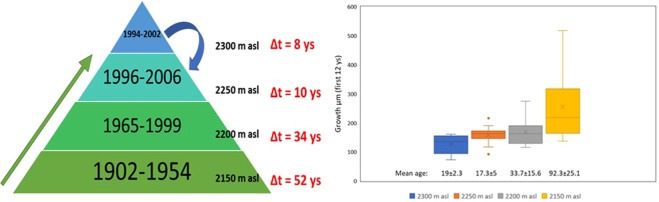
Left: a schematic representation of the distribution of ages at different altitudes. The age-range is shown in each “elevation band” and a time difference (Δ), as a residence time at a specific altitude, in upward or downward shift is reported. Arrows indicate the general tendency in treeline movement. Right: boxplot of the growth, during their first 12 years of life, of trees located at different altitudes at the treeline ecotone.

Tree basal area shows an expected significant negative correlation with both the year of birth of the trees (Kendall’s rank correlation τ = −0.72, P < 0.01; Fig. 6) and the altitude (τ = −0.69, P < 0.01). In contrast, mean annual growth is not significantly influenced by the altitude (τ = −0.23, P = 0.30; Fig. 6) and shows a hump-shaped tendency when plotted against the birth year of trees (quadratic function, R^2^ = 0.42). In fact, the groups of the oldest and youngest trees show a similar mean annual growth, which is lower than that of mid-age trees (Fig. 6). The positive-saturating relationship between the altitude and the birth year of trees (quadratic function, R^2^ = 0.85) shows detectable shifts as schematized in Fig. 7, where we find strong evidence that trees born before 1954 do not grow above 2,150 m and trees born before 1999 do not grow above 2,200 m a.s.l. The maximum altitude of 2,300 m a.s.l. was reached between 1994 and 2002, while after 2002 the youngest trees sampled (born around 2006) grow at a lower altitude of 2,250 m a.s.l. (Fig. 7).

To determine if the shape of the mean annual growth curve were a simple biological artifact or related to the changes in climatic conditions and establishment altitude, we compared only the first 12 years of growth of all sampled trees (e.g. we compared the first 12-years mean growth of trees born in 1900s vs. 1980s vs. 2000s). We show (Fig. 7, right plot) that the oldest trees (which are located, on average, at the lowest altitude on the treeline ecotone at ≈2150 m a.s.l.) grew more than mid-age and younger trees (located at medium [≈2200 m a.s.l.] and higher [2250–2300 m a.s.l.] altitudes, respectively).

**Figure 8:**
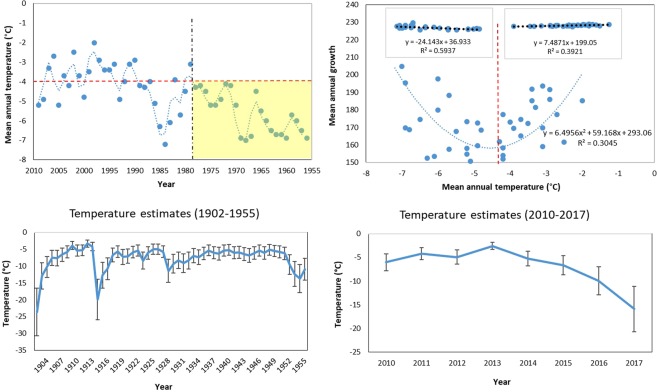
The variation of mean annual temperature from [ref.^29^] (left-upper panel) was related to the mean annual growth of the sampled trees and shows a hyperbolic curve (right-upper panel). This can be split in two linear trends at the −4.3 °C threshold (dashed red line) derived from the evidence that before 1978 (dashed black line) temperature never exceeded this value (yellow area). Therefore the estimated temperatures for the missing years in the observations by [ref.^29^] (lower panels) were derived from the linear regression equations shown in the small inserts of the growth-temperature plot (the dashed black lines represent the linearized trend after the split of the hyperbolic curve), at <−4.3 °C for 1902–1955 and >−4.3 °C for 2010–2017.

From a comparison between the variation of the mean annual temperature^29^ and our dendrochronological time-series (Fig. 8), we discovered that before 1978 the regional mean annual temperature remained below −4.3 °C. This threshold was, therefore, used to interpret the behavior of the hyperbolic curve (Fig. 8 upper-left panel) that emerged from the relationship between the mean annual growth and mean annual temperature (R^2^ = 0.34). This curve, at about −4.3 °C can be divided in two linear regressions (<−4.3 °C negative, R^2^ = 0.59; 4.3°C positive, R^2^ = 0.39), which allow an easier estimation of the missing annual temperature records (before 1956 and after 2009) to fill the gap of the available data in the literature for the area. The only long temperature record^29^ for the study-site covers a period between 1956 and 2009 and the most recent records (1966–2015)^31^ are from close meteorological stations but situated at lower altitudes.

The estimation for the years 1902–1955 (derived from the linear regression equation at <−4 °C) shows that temperature ranged between −5 °C and −10 °C, with some lower peaks between 1952–1955, 1926–1928, 1914–1916 and 1902–1904. The estimation for the years 2010–2017, shows a slight decreasing temperature trend, with a drop of 5–10 °C in the recent years (Fig. 8).

The results of the split sample test showed that the correlation coefficient and the product means for all calibration and verification periods were significant at P < 0.01 level. The sign tests were statistically significant in calibration and in the verification periods from 1956 and 1978 and from 1979 to 2009 at P < 0.05 level. The values of RE (0.86 and 0.77, respectively) and CE (0.32 and 0.36, respectively) were always positive, indicating that the regression was stable and reliable.

## Discussion and Conclusion

Similar to the patterns documented in other high-mountains of the world^4–6,10,11^, we found that the increase of temperature during the last century of climate change has led to an alteration of the tree growth trend and an upward shift until recently of the treeline in a remote preserved forest at the Aktru Research Station in the Altai Mountains. This can be attributed to the fact that areas with a harsh growing condition for woody species, previously covered by snow for most of the year, would become suitable for tree growth as the mean annual temperature increases, thus enabling a upward shift and expansion of the treeline ecotone^12^.

We showed that, as predictable, biometric (allometric) parameters, such as tree height, d.b.h. and crown width decrease with the altitude, with significant differences between trees in the dense treeline (at about 2,150 m a.s.l.) and those growing at higher elevation (up to 2,300 m a.s.l.). These differences, however, could be due not only to an upward shift of the new saplings but also to a differential growth trend related to altitudinal effects^50,51^. In fact, trees living at the limits of the treeline may show a reduced growth when subject to harder climatic conditions then their physiological limits (i.e. lower temperatures, frozen soil, stronger wind, etc.)^52^. To test if a variation in the tree growth trends and an upward shift of the treeline ecotone might be caused by increasing local and global temperatures, we further analyzed dendrochronological samples collected along some altitudinal transects. From an analysis of the complete dendrochronological time series from 1902 to 2017, we found a slight decrease in the general growth trend and relevant growth peaks for many years, all dated before 1968, when the mean annual temperature was always below the identified −4.3 °C threshold. In the last 50 years, trees did not show any sign of increased growth compared to the average trend for the century. This could be due to the fact that high-mountain trees are quite sensitive to low temperatures and grow more under a certain threshold (−4.3 °C in our study-site)^53–55^. In fact, comparing the available mean annual temperature observations from 1956 to 2009 with our dendrochronological time series, we detected that - in this time frame - the highest growth peaks (in 1952–1956, 1961–1962 and 1968) correspond (with a few years of lag) to the lowest temperature for the period. However, after 1968 (i.e. in the last 50 years except for 1983–1988, when the temperature dropped for a while and the tree growth temporarily increased), the temperatures were often above a −4.3 °C threshold, and the tree growth was constant (without any sudden growth peak) and slightly decreasing. The fact that the precipitation trend shows no change for the whole region in the last 50 years^31^ gives more weight to temperature importance and to its low extremes.

Our reconstructed temperature estimates for the period 1902–1955 derived from the dendrochronological time series, which was missing from the available observation on the local weather station^29,31^, well matched the periods of extreme cold temperatures in 1902–1906, 1913–1915, 1922–1923, 1926–1928 and 1950–1952 recorded by other biological and non-biological sources at comparable regional^56–58^ and continental scales^59,60^.

We also checked whether last-century temperature increase would have contributed to the shift of the treeline ecotone to higher altitudes. We showed that trees have actually had an accelerating upward movement during the last century, yet a recent slight downward movement. In fact, during the last 115 years, the “residence time” (intended as the years before the next 50-m upward shift) at an altitude of 2,150 m a.s.l. was approx. 52 years (from 1902 to 1954), then trees moved upward at about 2,200 m a.s.l. and kept growing at that elevation for approx. 34 years (from 1965 to 1999). Only from 1996 to 2006 (with a residence time of just 10 years) trees began growing at 2,250 m a.s.l. Some trees, between 1994 and 2002, reached even higher elevations and established at about 2,300 m a.s.l. (with a residence time at that altitude of only 8 years). This general trend in the upward shift of the treeline ecotone was relatively fast: after 1954, it took only 52 years for trees to move 150 m upward, whereas treeline “resided” for almost the same timespan (≈52 ys or even longer) at a lower elevation of 2,150 m a.s.l. Moreover, we did not find any tree born after 2002 established at the highest elevation point (2,300 m a.s.l.). The youngest sampled tree (which was born in 2006) resided 50 m below, which suggests a recent slight downward movement of the treeline likely due to the decrease of mean annual temperatures we detected for the last few years.

Our finding that young trees (approx. 17–19 years old, sampled at the higher altitudes) grow less than old trees (93 ± 25.21 years old, sampled at lower elevations), during their first 12 years of life, confirms a reduced tree growth at higher temperatures in the high-mountains^61^. In summary, this evidence suggests that the temperature increase during the past century may pose a double effect on mountain forests: in our study-site, tree growth decreased in recent years and the treeline ecotone moved 150 m upward in just about 50 years (after the 1960s), with a slight downward shift during the last decade (after 2000s).

In general, the rapid upward treeline shift that we detected combined with the reduced tree growth, may radically change the composition and landscape of the montane ecosystems. In areas on our study site where we did not recorded any trees, we found that a high percentage of the soil was covered by highly biodiverse meadows (with approx. 14 identified herb species, data not reported here) and by both economically and culinary valuable shrubs, including *Juniper spp*., *Vaccinium spp*. and *Ribes spp*. A continuous upward shift tendency, as the one documented for the last century in this study, would presumably lead to an expansion of the forest at the expense of meadow and shrub species, which would have no other area to migrate to once the mountain summit is reached^62^. On the other hand, we also identified a partial tendency inversion for the most recent decade of tree expansion at high elevations, which could represent a “temporary relief” for the survival of high-altitude meadow and shrub species. Trees did not move upward after 2004 and this might reflect the recent temperature decrease observed both at the local meteorological stations^31^ and from our estimates derived from the hyperbolic relationship between the mean annual growth and the 1956–2009 field-recorded temperatures. In fact, we found that as −4.3 °C represents a threshold between the negative growth tendency before the 1978 and the positive one after this year, estimated temperatures from 2010 to 2017 show a decreasing trend that, at least, could temporarily hinder the upward shift of the treeline. Further resources to reach and study these remote high-mountain areas would clarify whether this threshold of −4.3 °C applies to other treelines of the Altai Mountains.

We recognize that our study focuses on a specific area of the Russian part of the Altai Mountains and we need to be careful discussing the applicability our results to other areas of the region. However, the novel findings should not wait for publication for many years so that other sites can be analyzed but rather used to stimulate similar research at other similar ecosystems by immediate publication.

The scale of impacts of climate change in the Arctic and Alpine areas are of far more than academic importance and generalizations must be balanced by site-specific studies of relevance to local populations and local biodiversity hot-spots such as the Aktru high-mountains.

In conclusion, based on *in situ* treeline data and a dendrochronological analysis, we found an accelerating upward shift of treeline on a high-mountain vegetation ecotone until recently and a declining tree growth rate in this ecotone that are attributable to an increase in local air temperature in the past century. Our findings provide evidence of substantial ecological impacts of global climate change in high-mountain regions. Our study suggests that climate change, if left unabated, can cause dramatic changes in the structure, diversity, and landscape composition of high-mountain ecosystems such as the Altai Mountains.

## Methods

### Data sampling and wood core collection

After identifying a representative slope of the treeline ecotone in the mountain chain and checking from historical information and local people the absence of any relevant anthropogenic influence, a grid composed by 3 vertical (altitudinal) and 4 horizontal (at the same elevation) transects, at a distance of 50 m each, was drawn and placed on a georeferenced satellite map of the area in order to include the whole ecotone from the timberline, through treeline, to the isolated and highest individual trees of the tree species line (Fig. 1), whose definitions are conventions for communication and do not deserve a major scientific debate^49^. This sampling frame was subdivided in a 5 × 5 m grid in order to collect landscape data from the areas included in the frame but excluded from the sampling transects (Fig. 2). The coordinates of each transect and corners of the sampling frame were recorded with a 2-m precision.

Along each transect all trees with a diameter at the breast height (d.b.h.) >1 cm were identified at the species level, measured with a laser hypsometer-dendrometer (height, dbh and crown width) and georeferenced within the sampling grid (Fig. 2 and Table 1). Seedlings and saplings of trees (below the minimum height of 1.35 m and d.b.h. of 1 cm) were not measured but have been mapped and included in the category “Trees” (which represents all the non-sampled trees outside the transects) in Fig. 2 and Table 1. The coverage of the other vegetation composed by shrub and herb species (identified at the genus level) and of the main landscape features (big rocks and landslide areas) included in the sampling frame among transects was visually estimated and georeferenced within each cell of the grid (Fig. 2 and Table 1).

**Table 1:** Biometric (allometric) parameters at different altitudes (50 m range) and %Coverage of other vegetation types in the study-site.

	Altitude (m a.s.l.)		
	2150–2200	2200–2250 Mean ± SD (in cm)	2250–2300
Tree DBH	20.57 ± 11.91	15.16 ± 9.22	6.76 ± 6.97
Crown Width	435.51 ± 224.42	253.47 ± 70.28	251.1 ± 211.62
Tree Height	762.22 ± 356.52	397.87 ± 250.01	219.6 ± 72.49
	**Abundance (n° of individuals)**		
*Pinus sibirica*	45	15	8
*Larix sibirica*	10	0	1
Total trees	55	15	9
	**Coverage (100% = ~500 m** ^**2**^ **)**		
Meadow %	0	1	76
Shrubs %	19	39	0
Trees (non-sampled)%	11	6	1

At each intersection of the 3 vertical and 4 horizontal transects, the two closest trees were sampled with Haglöf increment borers in a cross design in order to collect two whole wood cores from each tree. This yielded to a collection of 103 individual trees from which 48 dendrochronological samples were extracted.

### Aerial photographs and 3-D modelling

In order to create a 3-D model of the study site, which could represent a reference for future studies of this remote area, 10 UHD aerial photographs taken by a professional drone from different angles where combined in order to create a three-dimensional mesh (Meshlab and Sketchup software) that allowed the reconstruction of 3-D aerial model. Then we georeferenced the 3-D model overlapping it to the 2-D Google Earth satellite image (Fig. 1). Unfortunately, no historical aerial photos of this treeline were available at both the local station and Tomsk State University.

### Biometric (allometric) data analysis

We tested a possible correlation between altitude and biometric (allometric) data with the Spearman’s rank correlation index at a significance level of α = 0.01 (R Development Core Team 2018). Then, we plotted a correlogram to show the correlation trends (R Development Core Team 2018).

The summary values per altitudinal ranges of the biometric data for all collected trees is shown in Table 1.

### Dendrochronological data analysis

The 48 wood cores collected were prepared following the common dendrochronological protocols and measured with a LINTAB measuring table with an accuracy of 0.01 mm, equipped with a Leica MS5 stereoscope. The tree-ring growth series were then visually (TSAPwin software, version 0.53^63^ and statistically (COFECHA^64,65^ cross-dated within trees and between trees (of the same site) for avoiding dating errors in the dataset. Then, an annual mean growth trend was calculated averaging the individual tree annual growth and a variance (SD) was attached (Fig. 4). The basal area of each sampled tree was also estimated.

We then detrended the tree-ring series (Fig. 5) through the estimation and removal of the tree’s natural biological growth trend, and standardized the detrended values by dividing each series by the growth trend to produce units in the dimensionless ring-width index (RWI). We used the most adopted methods available for detrending trough the package ‘detrendeR’ in R Development Core Team 2018. We implemented are a smoothing spline detrending via ffcsaps (*method* = “Spline”), a modified negative exponential curve (*method* = “ModNegExp”), a simple horizontal line (*method* = “Mean”), an “Ar” approach, and a “Friedman” approach.

The “Spline” approach uses a spline where the frequency response is 0.50 at a wavelength of 0.67 * “series length in years”. This attempts to remove the low frequency variability that is due to biological or stand effects.

The “ModNegExp” approach attempts to fit a classic nonlinear model of biological growth of the form f(t) = a exp(b t) + k, where the argument of the function is time^66^.

We also checked if a suitable nonlinear model could not be fitted (function is non-decreasing or some values are not positive) and we fitted a linear model. The “Mean” approach fits a horizontal line using the mean of the series. This method is the fallback solution in cases where the “Spline” or the linear fit (also a fallback solution itself) contains zeros or negative values, which would lead to invalid ring-width indices.

The “Ar” approach is also known as prewhitening where the detrended series is the residuals of an Ar model divided by the mean of those residuals to yield a series with white noise and a mean of one. This method removes all but the high frequency variation in the series and should only be used as such^61^.

These methods are chosen because they are commonly used in dendrochronology. There is a rich literature on detrending and many researchers are particularly skeptical of the use of the classic nonlinear model of biological growth (*f(t)* = *a exp(b t)* + *k*) for detrending^67^.

Finally, we detrended our time series with the “Friedman” approach that uses Friedman’s ‘super smoother’ as implemented in supsmu (R Development Core Team 2018).

We tested the significance of the time-series with a Mann-Kendall method, which is an effective test to detect the long-term change in time series^68^. In this study, this method was applied to detect the long-term trend change of the mean annual growth of the sampled trees.

**Figure 9:**
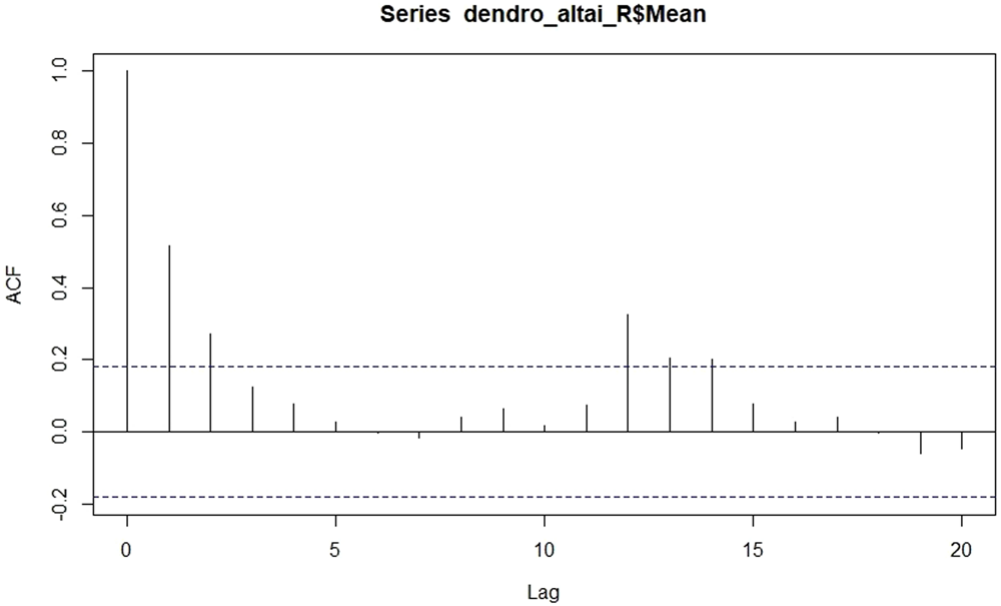
The autocorrelation function plotted against the different lags for the dendrochronological time-series.

**Figure 10:**
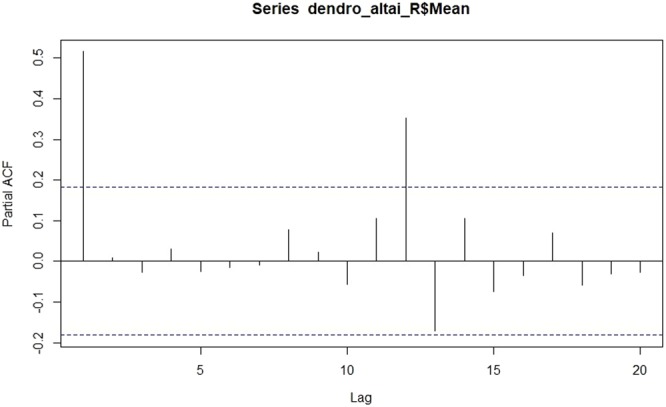
The partial autocorrelation function plotted against the different lags for the dendrochronological time-series.

Moreover, we checked the possible autocorrelation on our dendrochronological time-series (with an autocorrelation function, acf implemented R Development Core Team 2018). The resulting plot (Fig. 9) shows a significant correlation at lag 1 (≈1), lag 2 (≈0.5) and lag 13 (≈0.4) that decreases after a few lags. This pattern indicates an autoregressive term. Therefore, we used the partial autocorrelation function to determine the order of the autoregressive term (Fig. 10).

After accounting for the partial autocorrelation function we derived an additional curve based on this tree ring index (Fig. 4), which, however, does not differ much from the curves detrended with different methods (particularly from the Friedman’s curve). Most of the growth peaks and the general growth trend remained unaltered.

Finally, in order to evaluate the bias of our time series and the level of its accuracy (defined in terms of standard error) to sample estimates, we performed a block bootstrap analysis (tsboot package in R). This technique allowed an estimation of the bias of our distribution using random sampling methods.

**Figure 11:**
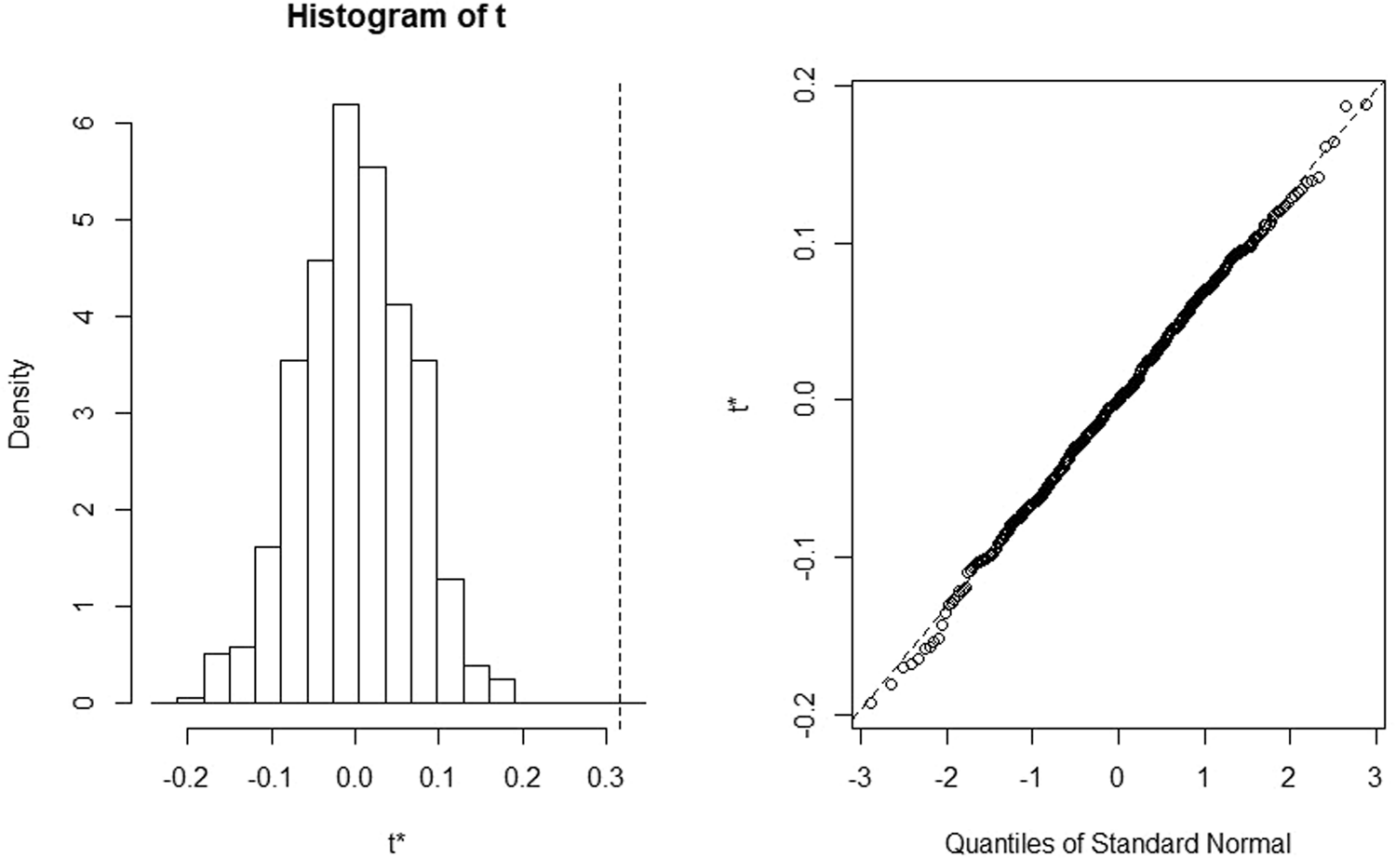
The histogram of the block bootstrapping method (left panel) shows the mean difference of bootstrapping compared to the observed difference (dashed vertical line) in the time series of the mean annual growth and the bias. The Q-Q plot of the 500 random bootstrapping with replacement (right panel) show the normality of the sample distribution.

We used a fixed block length of 1 to account for each lag of the time series and the results show a bias of −0.32 and a very low standard error of 0.06. We then plotted the histogram of the block bootstrapping method (Fig. 11), which shows that the mean difference of bootstrapping is well below the observed difference (dashed vertical line) in the time series of the mean annual growth (with the calculated bias of −0.32). The Q-Q plot of the 500 random bootstrapping with replacement shows that our time series has a normal distribution (Fig. 11).

### Dendrochronology vs. altitude, year of tree birth, and climate data analysis

We tested a possible correlation between our dendrochronological time series (mean annual growth and basal area) vs. altitude, year of tree birth, and climatic variations with the Kendall’s correlation index at a significance level of α = 0.01 (R Development Core Team 2018).

Because the relationship between the year of tree birth vs. both the altitude and the mean annual growth was non-linear, we applied a generalized linear model (glm package in R) and we considered a quadratic function and the related determination coefficients to describe these curvilinear relationships, in the mathematical form of:

1 $${\rm{y}}={{\rm{ax}}}^{2}+{\rm{bx}}\pm {\rm{c}}{\rm{.}}$$

To disentangle the curvilinear relationship between the mean annual growth and the year of tree birth, we analyzed the growth trends at the rarefied minimum age of all our core samples (12 years). In this way, we were able to compare the growth of the trees grouped per current age and altitude (4 groups at 2,150, 2,200, 2,250, 2,300 m a.s.l.) during their “youth”, in their first 12 years of life and to evaluate their growth variability.

Although some mean precipitation and temperature observations for the whole Altai region have been recently published^31^, we used the longest climatic time series available in the literature^29^, with a time span of 53 years (1956–2009). This was recorded from a meteorological station (that in Kosh-Agach) located at a comparable altitude (≈1,900 m a.s.l.) and location (linear distance ≈50 km) with respect to our study site.

Since, from the data in the literature, there is no evidence of a significant variability in the precipitation trends^29,31^, we considered only temperature records.

The mean annual temperature variability was correlated with the mean annual growth variability and a generalized linear model (quadratic function) was used to describe the relationship. Because the trend resulted in an almost hyperbolic curve (quadratic function), we inspected in any factor related to the predictor variable (temperature) could have influenced it. We discovered that a threshold of −4.3 °C was never passed before the 1978 and that this temperature approximately represents the turning point (close to the minimum point x = −b/2a of the eq. 1) of the curvilinear relationship between the mean annual growth and the mean annual temperature.

Therefore, to derive linear equations that would allow an estimation of the temperature during the missing years of observation from our dendrochronological time series, we divided the temperature-growth plot in two subplots (at −4.3 °C threshold). The parameter from the linear regression equation derived from the data points at a temperature <−4.3 °C were used to estimate the temperature for the period 1902–1955 (because of we show that the temperature was always below −4.3 °C before 1978) and those derived from the data points at a temperature >−4.3 °C were used to estimate the temperature for the period 2010–2017 (because the data points >−4.3 °C exclusively represents most of the growth trends after 1978).

Following a common approach to reconstruct temperatures from tree-ring records^69–71^, the stability and reliability of the regression Eq. 1 were assessed using the split sample method. We performed a validation by calibrating climate data from a sub-period by dividing the data into two parts: 1956–1978 and 1979–2009) and verifying the reconstruction using the remaining data. The results were evaluated by the correlation coefficient (r), the sign test (ST), the reduction of error test (RE), the coefficient of efficiency (CE) and the product means test (t) during the verification period. The actual and estimated data in year i of the verification period and the mean of the actual data in the calibration and verification periods were compared to calculate the sign of RE and CE following refs^72,73^, respectively.

We displayed the reconstructed curves of temperature variability and compared their general trends and selected relevant periods with the available information in the literature (see the Discussion section).

## References

[1] Easterling DR (1997). Maximum and minimum temperature trends for the globe. Science..

[2] Jeong SJ, Ho CH, Gim HJ, Brown ME (2011). Phenology shifts at start vs. end of growing season in temperate vegetation over the Northern Hemisphere for the period 1982–2008. Global Change Biology..

[3] Bathiany S, Dakos V, Scheffer M, Lenton TM (2018). Climate models predict increasing temperature variability in poor countries. Science advances..

[4] Banerjee K, Cazzolla Gatti R, Mitra A (2017). Climate change-induced salinity variation impacts on a stenoecious mangrove species in the Indian Sundarbans. Ambio..

[5] Rangwala I, Miller JR (2012). Climate change in mountains: a review of elevation-dependent warming and its possible causes. Climatic Change..

[6] Walther GR (2002). Ecological responses to recent climate change. Nature.

[7] Lenoir J, Gégout JC, Marquet PA, De Ruffray P, Brisse H (2008). A significant upward shift in plant species optimum elevation during the 20th century. Science..

[8] Gatti RC, Di Paola A, Bombelli A, Noce S, Valentini R (2017). Exploring the relationship between canopy height and terrestrial plant diversity. Plant Ecology..

[9] Gatti RC, Fath B, Hordijk W, Kauffman S, Ulanowicz R (2018). Niche emergence as an autocatalytic process in the evolution of ecosystems. Journal of Theoretical Biology..

[10] Walther GR, Beißner S, Burga CA (2005). Trends in the upward shift of alpine plants. Journal of Vegetation Science.

[11] Beckage B (2008). A rapid upward shift of a forest ecotone during 40 years of warming in the Green Mountains of Vermont. Proceedings of the National Academy of Sciences..

[12] Gatti RC (2016). The fractal nature of the latitudinal biodiversity gradient. Biologia..

[13] Alo, C.A., & Wang, G. Potential future changes of the terrestrial ecosystem based on climate projections by eight general circulation models. *Journal of Geophysical Research: Biogeosciences*. **113**(G1) (2008).

[14] Wolf A, Callaghan TV, Larson K (2008). Future changes in vegetation and ecosystem function of the Barents Region. Climatic Change..

[15] Rundqvist S (2011). Tree and shrub expansion over the past 34 years at the tree-line near Abisko, Sweden. Ambio..

[16] Beckage B (2008). A rapid upward shift of a forest ecotone during 40 years of warming in the Green Mountains of Vermont. Proceedings of the National Academy of Sciences..

[17] Kullman L (2007). Tree line population monitoring of Pinus sylvestris in the Swedish Scandes, 1973–2005: implications for tree line theory and climate change ecology. Journal of Ecology..

[18] Saxe H, Cannell MG, Johnsen Ø, Ryan MG, Vourlitis G (2001). Tree and forest functioning in response to global warming. New Phytologist..

[19] Pearson RG (2006). Climate change and the migration capacity of species. Trends in ecology & evolution..

[20] McLachlan JS, Clark JS, Manos PS (2005). Molecular indicators of tree migration capacity under rapid climate change. Ecology..

[21] Baker BB, Moseley RK (2007). Advancing treeline and retreating glaciers: implications for conservation in Yunnan, PR China. Arctic, Antarctic, and Alpine Research..

[22] Iverson LR, Prasad AM, Matthews SN, Peters M (2008). Estimating potential habitat for 134 eastern US tree species under six climate scenarios. Forest Ecology and Management..

[23] Holtmeier FK, Broll G (2005). Sensitivity and response of northern hemisphere altitudinal and polar treelines to environmental change at landscape and local scales. Global Ecology and Biogeography..

[24] Van Bogaert R (2011). A century of tree line changes in sub‐Arctic Sweden shows local and regional variability and only a minor influence of 20th century climate warming. Journal of Biogeography..

[25] Körner C (1998). A re-assessment of high elevation treeline positions and their explanation. Oecologia..

[26] Blyakharchuk TA, Wright HE, Borodavko PS, Van Der Knaap WO, Ammann B (2004). Late Glacial and Holocene vegetational changes on the Ulagan high-mountain plateau, Altai Mountains, southern Siberia. Palaeogeography, Palaeoclimatology, Palaeoecology..

[27] Sandel B (2011). The influence of Late Quaternary climate-change velocity on species endemism. Science..

[28] Cazzolla RG (2018). The last 50 years of climate‐induced melting of the Maliy Aktru glacier (Altai Mountains, Russia) revealed in a primary ecological succession. Ecology and Evolution..

[29] Politova NG, Sukhova MG, Zhilina TN (2013). Changes in the parameters of the temperature-humidity regime of the surface atmosphere and the reaction of mountain systems on the example of the Altai State Biosphere Reserve [in Russian]. Вестник Томского Государственного Университета..

[30] Mandych, А. F. *et al*. Biodiversity Conservation in the Russian Portion of the Altai-Sayan Ecoregion under Climate Change. *Adaptation Strategy*. Krasnojarsk**:** UNDP. 62 pp. (2012).

[31] Zhang D, Yang Y, Lan B (2018). Climate variability in the northern and southern Altai Mountains during the past 50 years. Scientific Reports..

[32] Sidorova OV (2013). The application of tree-rings and stable isotopes for reconstructions of climate conditions in the Russian Altai. Climatic Change..

[33] Xu G (2014). Relative humidity reconstruction for northwestern China’s Altay Mountains using tree-ringδ18O. Chines Science Bulletin..

[34] Zhang R (2016). Tree-ring-based moisture variability in western Tianshan Mountains since A.D. 1882 and its possible driving mechanism. Agricultural and Forest Meteorology..

[35] Zhang R (2017). Tree-ring-based precipitation reconstruction in southern Kazakhstan, reveals drought variability since A.D. 1770. International Journal of Climatology..

[36] Zhang R (2019). A tree ring-based record of annual mass balance changes for the TS.Tuyuksuyskiy Glacier and its linkages to climate change in the Tianshan Mountains. Quaternary Science Reviews..

[37] Esper J (2003). Temperature-sensitive Tien Shan tree ring chronologies show multi-centennial growth trends. Climate Dynamics..

[38] Ovtchinnikov D, Adamenko M, Panushkina I (2000). A 1105-year tree-ring chronology in Altai region and its application for reconstruction of summer temperatures. Geolines..

[39] Ning L, Liu J, Wang Z, Bradley RS (2018). Different influences on the tropical Pacific SST gradient from natural forcing and anthropogenic forcing. Int. J. Climatology..

[40] Peñuelas J, Canadell JG, Ogaya R (2011). Increased water‐use efficiency during the 20th century did not translate into enhanced tree growth. Global Ecology and Biogeography..

[41] Reed CC, Ballantyne AP, Cooper LA, Sala A (2018). Limited evidence for CO 2‐related growth enhancement in northern Rocky Mountain lodgepole pine populations across climate gradients. Global change biology..

[42] Bhatt US (2017). Changing seasonality of panarctic tundra vegetation in relationship to climatic variables. Environmental Research Letters..

[43] MacDonald GM, Kremenetski KV, Beilman DW (2008). Climate change and the northern Russian treeline zone. Philosophical Transactions of the Royal Society of London B: Biological Sciences..

[44] Rustad LEJL (2001). A meta-analysis of the response of soil respiration, net nitrogen mineralization, and aboveground plant growth to experimental ecosystem warming. Oecologia..

[45] Norby RJ (2005). Forest response to elevated CO_2_ is conserved across a broad range of productivity. Proceedings of the National Academy of Sciences..

[46] Battipaglia G (2015). Long tree-ring chronologies provide evidence of recent tree growth decrease in a central African tropical forest. PloS One..

[47] Grace J, Berninger F, Nagy L (2002). Impacts of climate change on the tree line. Annals of Botany.

[48] Xie Y, Sha Z, Yu M (2008). Remote sensing imagery in vegetation mapping: a review. Journal of Plant Ecology..

[49] Körner C, Paulsen J (2004). A world‐wide study of high altitude treeline temperatures. Journal of Biogeography..

[50] Vitasse Y, Delzon S, Bresson CC, Michalet R, Kremer A (2009). Altitudinal differentiation in growth and phenology among populations of temperate-zone tree species growing in a common garden. Canadian Journal of Forest Research..

[51] Way DA, Oren R (2010). Differential responses to changes in growth temperature between trees from different functional groups and biomes: a review and synthesis of data. Tree Physiology..

[52] Maxime C, Hendrik D (2011). Effects of climate on diameter growth of co-occurring Fagus sylvatica and Abies alba along an altitudinal gradient. Trees..

[53] Körner C (2003). Carbon limitation in trees. Journal of Ecology..

[54] Gedalof, Z. E. & Berg, A. A. Tree-ring evidence for limited direct CO_2_ fertilization of forests over the 20th century. *Global Biogeochemical Cycles*. **24**(3) (2010).

[55] Carrer M, Urbinati C (2006). Long‐term change in the sensitivity of tree‐ring growth to climate forcing in Larix decidua. New Phytologist..

[56] Kivinen S, Rasmus S, Jylhä K, Laapas M (2017). Long-term climate trends and extreme events in Northern Fennoscandia (1914–2013). Climate..

[57] Svyashchennikov, P. & Forland, E. Long-term trends in temperature, precipitation and snow conditions in Northern Russia. NMI Report Climate, 9 (2010).

[58] Jacoby GC (2000). Long-term temperature trends and tree growth in the Taymir region of northern Siberia. Quaternary Research..

[59] Pollack, H. N. *et al*. Surface temperature trends in Russia over the past five centuries reconstructed from borehole temperatures. *Journal of Geophysical Research: Solid Earth*. **108**(B4) (2003).

[60] Briffa KR (2008). Trends in recent temperature and radial tree growth spanning 2000 years across northwest Eurasia. Philosophical Transactions of the Royal Society of London B: Biological Sciences..

[61] Rossi S, Deslauriers A, Anfodillo T, Carrer M (2008). Age‐dependent xylogenesis in timberline conifers. New Phytologist..

[62] Walther GR, Beißner S, Burga CA (2005). Trends in the upward shift of alpine plants. Journal of Vegetation Science..

[63] Rinn, F. -*TSAPWin - Time Series Analysis and Presentation for Dendrochronology and Related Applications*, *Version 0*.*53*, «User Re- ference», Heidelberg. 91 pp (2005)

[64] Holmes RL (1983). Computer-assisted quality control in tree-ring dating and measurement. Tree-Ring Bulletin..

[65] Grissino-Mayer HD (2001). Evaluating crossdating accuracy: a manual and tutorial for the computer program COFECHA. Tree-Ring Research..

[66] Fritts, H. C. *Tree Rings and Climate*. Blackburn Press, Caldwell, NJ, USA, pp. 567 (2001).

[67] Cook, E. R. & Briffa, K. R. *Data analysis*. In: Cook E.R. & Kai- riukstis L.A. (Eds), «Methods of dendrochronology. Applications in the environmental sciences» Kluwer, Boston, 97–162 (1990).

[68] Burn DH, Hag EMA (2002). Detection of hydrological trends and variability. J. Hydro..

[69] Fritts, H. C. *Reconstructing large-scale climatic patterns from tree-ring data: t diagnostic analysis*. University of Arizona Press (1991).

[70] Cook ER, Meko DM, Stahle DW, Cleaveland MK (1999). Drought reconstructions for the continental United States. Journal of Climate..

[71] Liu Y (2015). A tree‐ring‐based June–September mean relative humidity reconstruction since 1837 from the Yiwulü Mountain region, China. International Journal of Climatology..

[72] Lorenz, E. N. *Empirical orthogonal functions and statistical weather prediction*. *Statistical Forecasting*. Scientific Rep. 1, Department of Meteorology, Massachusetts Institute of Technology, Cambridge, MA, 57 pp (1956).

[73] Nash JE, Sutcliffe JV (1971). Riverflow forecasting through conceptual models 1, A discussion of principles. J. Hydrol..

